# Prospective comparison of robotic versus laparoscopic one-anastomosis gastric bypass: real-life analysis of procedural standardization and safety during the initial adoption phase

**DOI:** 10.1007/s11701-026-03195-x

**Published:** 2026-02-10

**Authors:** Nicolas Zucchini, Silvia Puddu, Enrico Moroni, Giovanni Fantola

**Affiliations:** 1 Department of Medical, Surgical and Health Sciences, University of Trieste, Trieste, Italy; 2 Metabolic and Obesity Surgery Unit, ARNAS G. Brotzu, Cagliari, Italy; 3 Department of Surgery, University of Cagliari, Azienda Ospedaliero-Universitaria, Presidio Policlinico di Monserrato, Monserrato, Italy

**Keywords:** Bariatric and metabolic surgery, Robotic surgery, OAGB, Learning curve, DaVinci

## Abstract

The adoption of robotic One-Anastomosis Gastric Bypass (rOAGB) is increasing, yet comparative data with the established laparoscopic approach remain limited. This study evaluates the initial adoption phase of rOAGB by a single surgeon, focusing on procedural consistency and safety compared to a laparoscopic (LAP) team. We prospectively analyzed 135 consecutive patients undergoing primary OAGB between October 2024 and October 2025. 81 LAP procedures (LAP group) were compared to 54 robotic procedures (rOAGB group). The rOAGB cohort represented the first series of this specific procedure for a surgeon experienced in other robotic platforms. The primary endpoint was procedural standardization, measured by operative time (OT) variance. Secondary endpoints included 30-day morbidity. The rOAGB series was chronologically divided into two six-month periods based on the study’s one-year duration. This temporal split resulted in an equal distribution of cases (27 procedures per semester). Baseline characteristics were comparable (*p* > 0.05). LAP was significantly faster (mean OT 86.0 ± 26.6 min vs. 96.8 ± 20.8 min; *p* = 0.004). However, rOAGB demonstrated a significantly narrower distribution of OTs (SD 20.8 vs. 26.6). In the rOAGB cohort, an F-test revealed a significant reduction in OT variance between the first and second semester of activity (*p* = 0.0134), indicating rapid stabilization. 30-day complication rates were similar (1.2% LAP vs. 3.7% rOAGB; *p* = 0.56). While LAP surgery remains faster, the robotic platform facilitates superior procedural consistency during the adoption phase. The robotic approach achieved significant standardization within 27 cases, maintaining a safety profile comparable to the established LAP technique.

## Introduction

 Metabolic and bariatric surgery (MBS) is constantly growing worldwide, as is Robotic Surgery [1]. One Anastomosis Gastric Bypass (OAGB) is currently the third most common procedure worldwide due to its metabolic efficacy and safety profile [2, 3]. It combines a metabolic action thanks to the increase in Gastric Inhibitory Peptide (GIP) and glucagon-like peptide 1 (GLP-1) levels, similar to Roux-en-Y Gastric Bypass (RYGB) [4–6], and a hypoabsorptive effect, both given by the direct arrival of food in the terminal jejunum- ileum. With these effects together, OAGB can lead to a higher percentage of excess weight loss (%EWL) maintaining safety in the long term, compared to Sleeve Gastrectomy (SG) and RYGB in different studies [7]. While the safety of robotic RYGB (rRYGB) and robotic SG (rSG) is well-documented [8, 9], there is a critical gap in literature regarding the transition to robotic OAGB (rOAGB). A common challenge in introducing robotics is the “learning curve” (LC), traditionally measured by a reduction in operative time (OT). However, we propose a shift in this paradigm: evaluating the LC through procedural standardization—the ability to achieve consistent, predictable results regardless of initial speed.

This study aims to compare the robotic and laparoscopic (LAP) approaches in a single-center experience. The primary objective was to evaluate the difference in OT and, specifically, to quantify procedural standardization. Secondary objectives included the assessment of 30-day postoperative outcomes to ensure non-inferiority during the adoption phase.

## Methods

### Study design and patient population

This prospective observational study included 135 consecutive patients scheduled for OAGB at ARNAS G. Brotzu, Cagliari, between October 2024 and October 2025. Patients were allocated to either the robotic or LAP group based on the availability of the robotic platform.

The rOAGB procedures (*n* = 54) were performed by a single senior surgeon (G.F.) with extensive experience in LAP bariatric surgery (> 500 cases) and general robotic surgery (> 100 cases). Crucially, this series represents the surgeon’s initial adoption phase specifically for the OAGB procedure on the robotic platform. The LAP procedures (*n* = 81) were performed by a team consisting of the same senior surgeon and one junior surgeon (N.Z.) undergoing training.

Preoperative data included age, sex, weight, height, body mass index (BMI), and comorbidities. While all patients followed a standardized institutional Enhanced Recovery After Surgery (ERAS) [12] protocol (aiming for discharge on the second postoperative day), the actual length of stay (LOS) was recorded to account for deviations due to complications.

## Surgical technique: robotic OAGB (rOAGB)

The procedure is performed entirely robotically using the da Vinci Xi Surgical System. The patient is placed in a supine, reverse Trendelenburg position. Pneumoperitoneum is induced with a Verres needle in the left hypochondrium (Palmer’s point) at 12 mmHg.

**Fig. 1:**
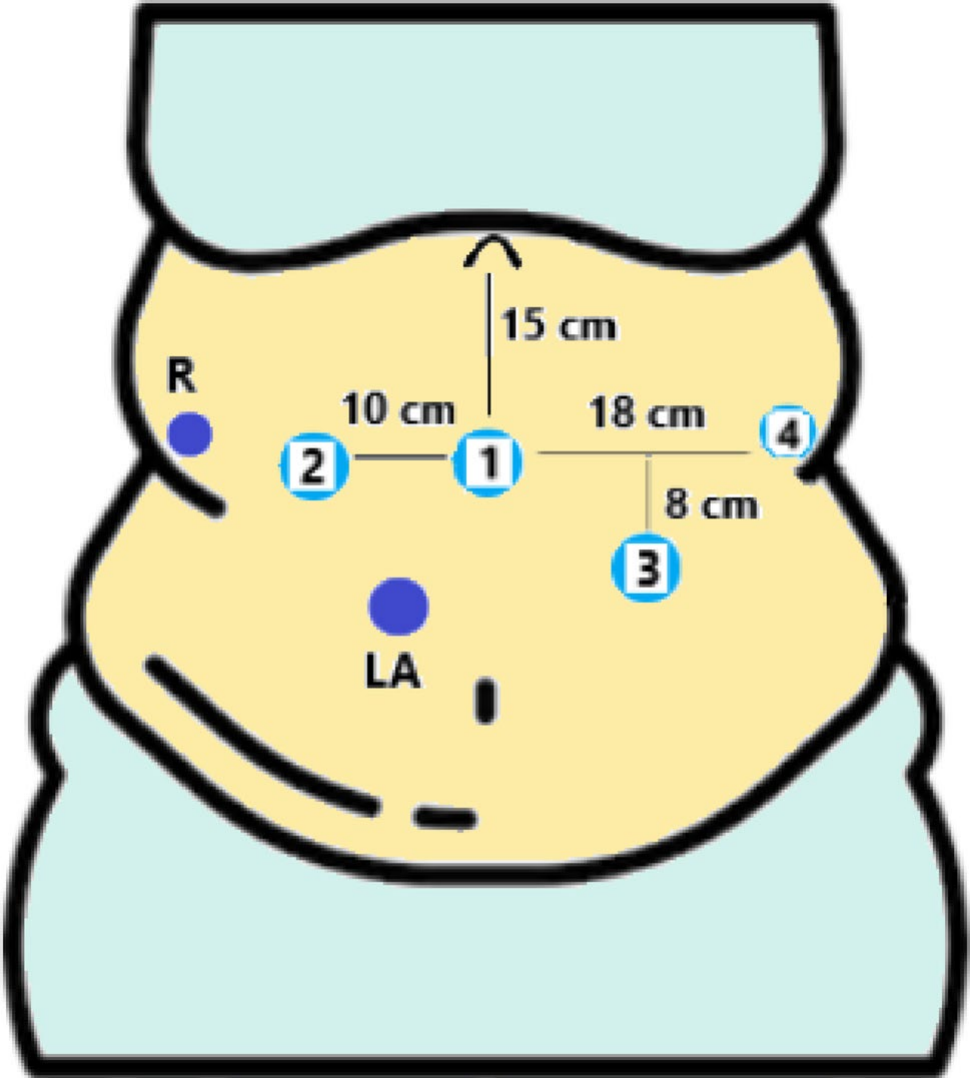
Standardized Robotic Setup. Schematic representation of the trocar configuration and port placement for the robotic One-Anastomosis Gastric Bypass (rOAGB). The layout depicts the positioning of the robotic ports relative to the assistant ports, designed to optimize triangulation and ergonomics

Trocars are positioned as showed in Fig. 1: a camera port is placed 15 cm below the xiphoid process in the epigastrium. Robotic trocar for arm 2 is placed 10 cm on the right in right flank; robotic trocar for arm 4 is placed 18 cm on the left in the left flank, all on the same line. Robotic trocar for arm 3 is placed between the camera port and arm 4, on a line which is 8 cm below. Two LAP assistant ports are used: a 5 mm trocar in the right hypochondrium for the liver retractor and a 12 mm accessory trocar between arm 1 and the camera port, on the same line of arm 3.

After a preliminary abdominal exploration, the ligament of Treitz is identified, and the biliopancreatic limb is measured to a length of 180 cm laparoscopically. The robotic system is then docked.

The procedure begins with the creation of the gastric pouch. The lesser curvature is opened near the *pes anserinus* (“crow’s foot”), followed by a transverse division of the stomach using a 60 mm robotic linear stapler (Sureform ^®^, blue cartridge). A long, narrow gastric pouch is then created along a 40 Fr bougie using sequential firings of 60 mm staplers (Sureform ^®^ blue and white cartridges). It is important to follow the posterior tunnel, which lands medially to the posterior gastric vessel (when present) and go in the direction of the left bundles originating from the right pillar of the diaphragm, which delimit the esophageal hiatus. Following this way is possible to achieve safety, limiting the risk of spleen injury, and efficacy maintaining the right distance from His’ angle.

A side-to-side anterior gastrojejunal anastomosis is fashioned using the robotic linear stapler (white cartridge). The resulting common enterotomy is closed with a continuous 3-0 barbed suture (Stratafix). A key step of our technique involves creating an anti-reflux suture: three stitches are placed between the biliopancreatic limb and the gastric pouch using a 3-0 barbed suture to prevent biliary reflux. An intraoperative leak test is then performed with methylene blue and air bubble test, which must be negative. A final inspection of the anastomosis and hemostasis is performed. Mesenteric defects are not closed at the end of the procedure. The robotic system is undocked, and the trocars are removed under direct vision. The port sites are then closed in layers.

## Laparoscopic technique

Patients and trocars are positioned in a similar way like rOAGB. After pneumoperitoneum with Verres needle, a camera port of 12 mm is placed 15 cm below the xiphoid process in the epigastrium, another two trocar (12 mm) on the same line, on the right and the left; two assistant trocars of 5 mm in the right and left ipocondrium. The procedure is performed using the exact same methods and steps as the rOAGB described above, including the length of the biliopancreatic limb and the choice of linear stapler cartridges used for creating the gastric pouch and the gastrojejunal anastomosis. Mesenteric defects are not closed at the end of the procedure.

### Statistical analysis

Continuous variables were expressed as mean **±** standard deviation (SD) and median. Normality was assessed using the Shapiro-Wilk test. Baseline characteristics were compared using Welch’s t-test for continuous variables and the Chi-square or Fisher’s exact test for categorical variables.For the primary endpoint, mean operative times (OT) were compared using an independent sample t-test. To assess procedural standardization, two analyses were performed: distribution analysis and variance comparison.

For what concerns distribution analysis OT for both groups were plotted as Gaussian curves to visually represent variability. To quantify the LC, the rOAGB group was chronologically divided into two six-month periods based on the study’s one-year duration. This temporal split resulted in an equal distribution of cases (27 procedures per semester), allowing for a balanced comparison of OT variance between the initial and subsequent adoption phases An F-test was used to compare the variance in OT between these periods. An exploratory analysis of the LAP cohort compared OTs between the senior and junior surgeons using Welch’s t-test. A two-tailed p-value < 0.05 was considered statistically significant. All analyses were performed using Google Sheets and statistical software.

## Results

A total of 135 patients were enrolled, in the same period, in this prospective non-randomized study: 81 (60%) in the LAP group and 54 (40%) in the rOAGB group. The baseline demographic and clinical characteristics of both cohorts are summarized in Table 1. There were no statistically significant differences between the two groups in terms of age, sex, BMI, or any measured comorbidities. The two cohorts were thus considered comparable for this analysis.

**Table 1:** Demographic and Clinical Characteristics. Comparison of baseline demographic data and preoperative comorbidities between the Laparoscopic (LAP) and Robotic (rOAGB) cohorts

Characteristic	Laparoscopic	Robotic	*p*-value
Age (years)	46.41 ± 11.63	45.93 ± 11.71	0.815
Sex: M (n, %)	11 (13.6%)	7 (13.0%)	1.000
Sex: F (n, %)	70 (86.4%)	47 (87.0%)	
BMI	44.92 ± 6.76	43.10 ± 6.20	0.109
Diabetes type 2 (n, %)	16 (19.8%)	7 (13.0%)	0.427
OSAS (n, %)	8 (9.9%)	8 (14.8%)	0.550
Hypertension (n, %)	34 (42.0%)	21 (38.9%)	0.858
GERD (n, %)	18 (22.2%)	13 (24.1%)	0.967
Osteoarticular disease(n, %)	23 (28.4%)	12 (22.2%)	0.548
Dyslipidemia (n, %)	24 (29.6%)	14 (25.9%)	0.785
Revision Surgery (n, %)	5 (6.2%)	0	0.055

Data are presented as mean ± standard deviation for continuous variables and as frequency (percentage) for categorical variables. Statistical significance was set at *p* 0.05. Abbreviations: *SD* Standard Deviation, *BMI* Body Mass Index, *OSAS* Obstructive Sleep Apnea Syndrome, *GERD* Gastroesophageal Reflux Disease

Analysis of the primary endpoint revealed a statistically significant difference in OT. The LAP group was, on average, 10.86 min faster than the rOAGB group (86.01 ± 26.66 min vs. 96.87 ± 20.88 min, *p* = 0.0045). The median times also reflected this difference (80.0 min for LAP vs. 95.0 min for rOAGB), as detailed in Table 2.

**Table 2:** Intraoperative Parameters. Comparative analysis of operative times, detailing mean, median, and standard deviation values. Operative time is defined as skin-to-skin duration

**Intervention Type**	**Mean OT**	**Median OT**	**SD OT**	**p-value**
Laparoscopic (n=81)	86.01	80.0	26.66	
Robotic (n=54)	96.87	95.0	20.88	
				0.0045

Abbreviations: *OT* Operative Time, *SD* Standard Deviation

**Fig. 2:**
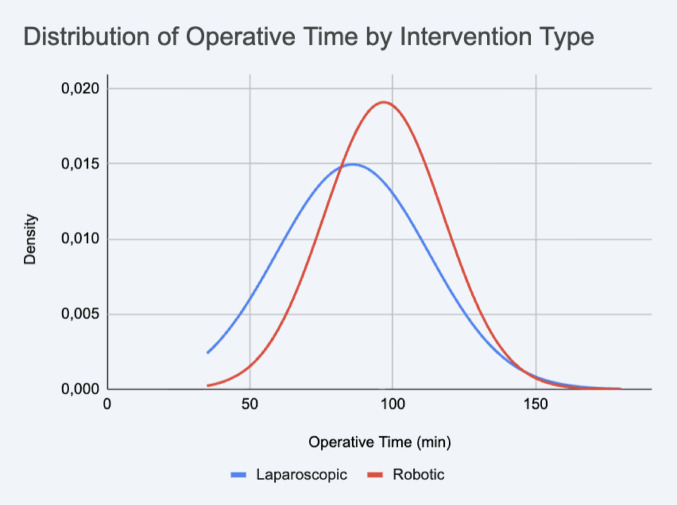
Operative Time Comparison. Comparative analysis of operative times between the Laparoscopic (LAP) and Robotic (rOAGB) groups. The graph illustrates the distribution of operative times; a higher curve peak corresponds to a lower standard deviation, indicating greater procedural consistency and reproducibility in the robotic group

The visual comparison of the distributions in Fig. 2 confirms these statistical findings. The laparoscopic cohort (blue curve) displays a distribution shifted to the left (faster) but is visibly wider and flatter, indicating higher variability. Conversely, the rOAGB cohort (red curve) is taller and narrower, suggesting higher predictability.

To investigate the source of the high variability within the LAP group, an exploratory analysis was performed comparing the 70 cases performed by the senior surgeon (G.F.) against the 11 cases performed by the junior surgeon (N.Z.). The results are detailed in Table 3. A statistically significant difference was found in the mean OT (84.68 min for senior vs. 95.50 min for junior, p = 0.042). Notably, the SD for the senior surgeon was nearly triple that of the junior surgeon (27.90 vs. 12.12), confirming the junior surgeon’s cases were highly consistent and predictable, while the senior surgeon’s were highly variable.

**Table 3:** Operative Time Analysis within the Laparoscopic Cohort. Assessment of operative time (OT) variability stratified by surgeon seniority (Senior vs. Junior)

LAP Surgeon	N. of Cases	Mean OT (min)	SD OT (min)	p-value
Total LAP cases	81	86.01	26.66	
Senior (F.G.)	70	84.68	27.90	
Junior (Z.N.)	11	95.50	12.12	
				0.042

Abbreviations: *LAP* Laparoscopic, *SD* Standard Deviation

To assess the LC and standardization of the robotic technique, the rOAGB cohort (n=54) was analyzed by dividing the one-year study period into two equal six-month halves. This chronological division resulted in 27 cases per period. The mean OT showed a tendency to decrease, from 99.11 ± 25.38 minutes in the first semester to 94.63 ± 15.31 minutes in the second semester, although this difference was not statistically significant (p = 0.43). 

**Fig. 3:**
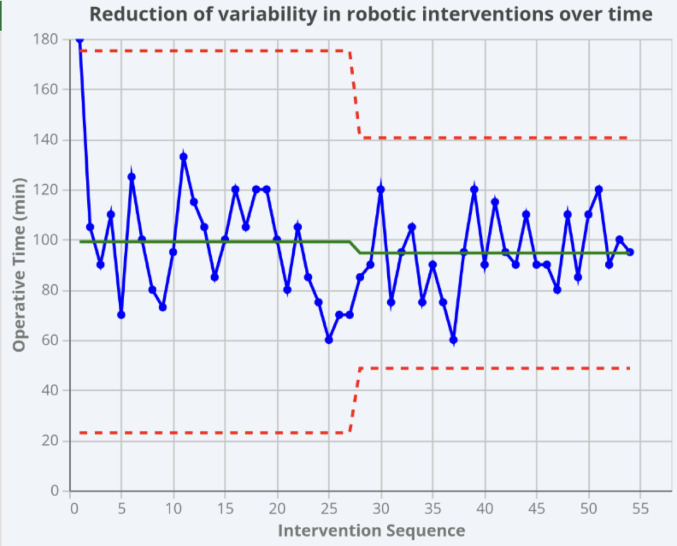
Reduction of Variability in Robotic Interventions. Temporal analysis of operative time variability within the robotic cohort. The trend demonstrates a significant reduction in variability (standard deviation) after overcoming the initial adoption phase (defined as the first 27 cases), highlighting the acquisition of procedural standardization

 In contrast, the analysis of variability confirmed this split-point: an F-test revealed a statistically significant reduction in variance between the first 27 and the last 27 cases (p = 0.0134). This indicates that the LC for procedural standardization was achieved after the first six months of activity, after which the procedure became significantly more consistent and predictable. Fig. 3

**Secondary endpoints**, including 30-day postoperative outcomes, are presented in Table 4.

**Table 4:** 30-Day Postoperative Outcomes. Comparative analysis of early complications (classified according to the Clavien-Dindo system), readmissions, and reinterventions between the Laparoscopic and Robotic cohorts

Approach	Complications (n; %)	Clavien Dindo	Readmissions (n; %)	Reinterventions (n; %)	p-value
Laparoscopic	1; 1.23%	II	0; 0%	0; 0%	
Robotic	2; 3.70%	I, IIIb	0; 0%	1; 1,92%	
					0.56

Data are presented as number and percentage

The overall morbidity rate was low. The LAP group experienced one Clavien-Dindo II complication (1.2%). The rOAGB group experienced two complications (3.7%): one Clavien-Dindo I, and one Clavien-Dindo IIIb complication which required surgical re-intervention, resulting in a 10-day length of stay. As confirmed by Fisher’s Exact Test, there was **no statistically significant difference** in the overall complication rates between the two groups (**p = 0.564**).

## Discussion

This study prospectively compared the initial adoption phase of robotic rOAGB with the established LAP technique in a single-center setting. Consistent with previous literature regarding robotic RYGB and SG [9, 13], our results confirm that rOAGB OT was significantly longer than laparoscopy. While the LAP approach was statistically faster, the primary advantage of the robotic platform manifested as a rapid achievement of procedural standardization, aligning with findings in general robotic surgery [14]. The finding that laparoscopy was, on average, 10.8 minutes faster (p=0.0045) is consistent with early reports on robotic surgery introduction [9]. However, we argue that this difference, although statistically significant, lacks clinical relevance.

A common criticism of robotics is the perceived increase in OT; yet, our data suggest this “cost” is minimal and offset by a superior level of procedural consistency, even during the initial adoption phase [15]. The rOAGB cohort demonstrated significantly lower overall variability (SD 20.88) compared to the LAP cohort (SD 26.66), a trend visually represented by the narrower Gaussian distribution in Fig. 2. This finding is particularly robust when considering the operator heterogeneity in the LAP group.

Our data suggests that the robotic platform acts as a"procedural equalizer." As articulated by Azagra, Pascotto, et al., laparoscopy often involves "many hands and many minds," whereas robotics is characterized by "many minds but one hand—that of the surgeon leader" [16]. LAP surgery is intrinsically teamwork-dependent, relying on the assistants’ skills to achieve optimal results. This dependency introduces procedural variability, which is minimized by the robotic platform where a single operator controls all critical aspects of the procedure (e.g., retraction, camera, and dissection). Furthermore, our analysis of the 54 robotic cases demonstrates that this standardization was achieved rapidly. While surgical speed did not significantly increase (p = 0.43), the procedural variance was significantly reduced (p = 0.0134) between the first and second halves of the series. This indicates that the first six months of adoption, corresponding to a 27-case LC, were sufficient for an experienced surgeon to adapt a known procedure to a new platform and achieve high predictability [17]. This supports the question posed by the Italian Robotic community [1] regarding whether robotic platforms facilitate a safer and more efficient LC compared to laparoscopy.

Regarding safety, 30-day outcomes were statistically comparable (p=0.564), consistent with literature on rRYGB and rSG [10, 11, 18, 19]. Notably, the single major complication in the rOAGB group (a Clavien-Dindo IIIb) occurred within the first 27 cases, coinciding with the period of higher variability (p=0.0134). This reinforces the concept that procedural risk is highest during the early, non-standardized phase of LC.

An emerging benefit of the robotic platform is its role as a modern training tool [20]. The junior surgeon’s role as a bedside assistant provides a unique, immersive perspective on standardized procedural steps. This exposure may explain the high consistency (low SD) observed in the junior surgeon’s limited LAP series (n=11), suggesting a cross-platform educational benefit that warrants further investigation.

We must acknowledge several limitations. The study is non-randomized, and the comparison involves a heterogeneous LAP team (including senior and junior surgeons) versus a single-operator robotic series. Furthermore, the LAP group included a small cohort of revisional procedures (6.2%), which inherently increases variance. However, we argue that this heterogeneity strengthens our conclusions: even when compared to a multi-operator "real-life" LAP practice that handles complex cases, the robotic platform provided a level of predictability and standardization that was evident from the earliest stages of adoption. Finally, this study only assesses 30-day outcomes; future research should focus on whether this procedural standardization leads to improved long-term metabolic and nutritional results.

## Conclusion

In conclusion, this prospective study demonstrates that while rOAGB requires a statistically significant increase in OT compared to the multi-operator LAP approach, this difference is offset by a superior level of procedural standardization. The mean increase of 10.8 minutes (p = 0.0045) is counterbalanced by the rapid achievement of high execution consistency, with a significant reduction in OT variance (p = 0.0134) observed after the first six months of adoption, corresponding to the 27th case. By allowing the surgeon to directly control every critical phase of the procedure, the robotic platform acts as a procedural equalizer, minimizing the variability inherent in LAP surgery, which remains strictly dependent on teamwork and the coordination between the lead surgeon and assistants. This enhanced predictability supports a 30-day safety profile comparable to the established LAP technique (p = 0.564), with major complications occurring exclusively during the initial, non-standardized phase of the LC. Beyond the primary surgeon’s performance, the robotic platform emerges as an effective educational tool, as the standardized visualization and maneuvers provided to the bedside assistant may accelerate the LC for junior surgeons. Ultimately, rOAGB is confirmed as a safe and highly reproducible technique that prioritizes consistency and precision; however, further studies with long-term follow-up are necessary to determine if this procedural standardization translates into improved metabolic and nutritional outcomes for the patient.

## Data Availability

The datasets generated during and/or analysed during the current study are available from the corresponding author on reasonable request.
